# Reversal of Roux-en-Y Gastric Bypass to Normal Anatomy in a Severely Malnourished Patient After Metabolic Bariatric Surgery

**DOI:** 10.7759/cureus.71240

**Published:** 2024-10-11

**Authors:** Rajdave Singh Sadu Singh, Guo Hou Loo, Guhan Muthkumaran, Aishath Azna Ali, Nik Ritza Kosai

**Affiliations:** 1 Upper Gastrointestinal and Metabolic Surgery Unit, Department of Surgery, Hospital Canselor Tuanku Muhriz, Universiti Kebangsaan Malaysia, Kuala Lumpur, MYS

**Keywords:** diabesity, metabolic syndrome, refeeding syndrome, rygb complications, rygb surgery

## Abstract

Obesity stands as a prominent health challenge in our society, with metabolic bariatric surgery (MBS) emerging as a solution due to its efficacy in addressing obesity-related type 2 diabetes mellitus (T2DM). Roux-en-Y gastric bypass (RYGB) and one-anastomosis gastric bypass (OAGB) remain the most common MBS after sleeve gastrectomy. Complications from RYGB are uncommon but include anastomotic stricture, marginal ulcers, small bowel obstruction, and nutritional complications.

We present a 52-year-old lady with an initial body mass index (BMI) of 27.6 kg/m^2^ and poorly controlled T2DM who presented with generalized body weakness and uncontrolled weight loss after an RYGB performed four months earlier. She was cachexic with a BMI of 17 kg/m^2^,with generalized anasarca with a multitude of electrolyte disturbances. After nutritional optimization, she underwent a reversal surgery back to normal anatomy.

Reversal of RYGB to normal anatomy is a complex surgical procedure and is often the last resort undertaken in patients experiencing severe complications from the initial surgery. Indications include malnutrition, severe dumping syndrome, excessive weight loss, and recalcitrant marginal ulcers. Our case outlines the importance of proper patient selection for MBS and highlights the preoperative management of RYGB reversal to normal anatomy. We also describe the surgical procedure using a stepwise approach.

In conclusion, the reversal of RYGB to normal anatomy should only be undertaken after a careful period of prehabilitation to reduce perioperative complications. The inclusion of dietitians, endocrinologists, and physiotherapists is crucial to ensure the best possible outcome.

## Introduction

Obesity stands as a prominent health challenge in our society, with bariatric surgery emerging as a solution due to its efficacy in addressing obesity, hypertension, and diabetes. The intertwining of obesity and diabetes exacerbates morbidity and mortality rates, amplifying the economic burden on healthcare systems. Projections indicate that the cost of diabetes could soar to nearly $500 billion by 2030 underscoring the urgency for effective treatment strategies and sustained remission [1]. Roux-en-Y gastric bypass (RYGB) surgery is a widely performed bariatric procedure involving the creation of a small pouch from the stomach and its connection with the jejunum. This procedure is medically recommended for obese individuals with a body mass index (BMI) exceeding 35 alongside type 2 diabetes mellitus (T2DM) or a BMI of 30 with unsuccessful attempts at weight loss [2]. 

In 2022, the American Society for Metabolic and Bariatric Surgery and the International Federation for the Surgery of Obesity and Metabolic Disorders (IFSO) published a major upgrade that recommended bariatric surgery for Asians with a BMI of ≥27.5 kg/m^2^ [3].

In Malaysia, according to the National Health and Morbidity Survey (NHMS) 2023, approximately 54.4% of the population is obese; this is an increase of 10% from 44.5% in 2011 [4]. Alongside this, diabetes is projected to affect seven million Malaysian adults aged 18 and older by 2025, posing a major public health risk with a diabetes prevalence of 31.3% [5]. According to local guidelines, recommendations were made based on the Malaysian Bariatric and Metabolic Working Committee's consensus statement where bariatric surgery is recommended for patients with a BMI of >32.5 kg/m^2^ with metabolic syndrome or cardiovascular risk and patients with a BMI of 37.5 without such comorbidities [6]. According to the Eighth IFSO Global Registry report, sleeve gastrectomy remains the most popular metabolic bariatric surgery (MBS) globally followed by RYGB and one-anastomosis gastric bypass (OAGB); however, nine registries reported RYGB, OAGB, and other procedures more often than sleeve gastrectomy [7]. Our local committee consensus recommends four procedures: restrictive (gastric band, sleeve gastrectomy) and malabsorptive procedure (biliopancreatic diversion/duodenal switch, RYGB). In this context, we present the case report of a 52-year-old woman experiencing severe malnutrition following RYGB surgery in her native country.

## Case presentation

A 52-year-old woman underwent RYGB as a metabolic bariatric surgery in her native country due to poorly controlled T2DM. She was on a high dose of insulin prior to surgery. At the time of the index surgery, her BMI was 27.6 kg/m² (weight 58 kg, height 145 cm). The index surgery was uneventful, and her HbA1c improved post-surgery from 9% to 4%, pre-surgery. However, after four months post-surgery, she experienced progressive rapid weight loss, and subsequently, she began experiencing weakness and reduced oral intake, accompanied by vomiting and frequent passage of loose stools. She initially sought medical attention in her native country where she was diagnosed to have inflammatory bowel disease and was started on mesalamine. Due to worsening symptoms, she subsequently presented to our center.

Upon arrival at our hospital, the patient was in a poor physical state, weighing only 37 kg with a BMI of 17 kg/m². She complains of oral intolerance and asthenia. On physical examination, she has anasarca with temporal muscle wasting. Otherwise, her cardiopulmonary and abdominal examinations were unremarkable. Relevant laboratory studies are included in Table 1.

**Table 1:** Relevant laboratory parameters for the patient on admission

Lab parameters	Values	Reference range
Hemoglobin	7.6 g/dl	12-16 g/dl
Platelet count	256,000/mcL	150,000-400,000/mcL
Serum albumin	19 g/L	34-54 g/L
Serum magnesium	0.58 mmol/L	0.85-1.10 mmol/L

Due to her inability to tolerate oral intake, she was started on parenteral nutrition. Initially, total parenteral nutrition was started at 10 kcals/kg actual body weight/day. It was started in a cyclical manner to ensure the patient was able to ambulate during the day.

Subsequently, parenteral nutrition was increased to 12 kcals/kg actual body weight/day with the addition of multivitamins. During this increment, we noted the patient developed early refeeding syndrome evident by hypomagnesemia. Upon resolution of her refeeding syndrome, parenteral nutrition was further increased to 25 kcals/kg actual body weight/day. As her condition improved further, enteral feeding was established with the commencement of nourishing fluid and then a soft diet. Concurrently, while optimizing her nutritional status, she underwent esophagogastroduodenoscopy, colonoscopy, and an echocardiogram as part of the preoperative assessment, all of which were normal. As part of prehabilitation, she also underwent daily physiotherapy sessions for limb-strengthening exercises.

**Figure 1:**
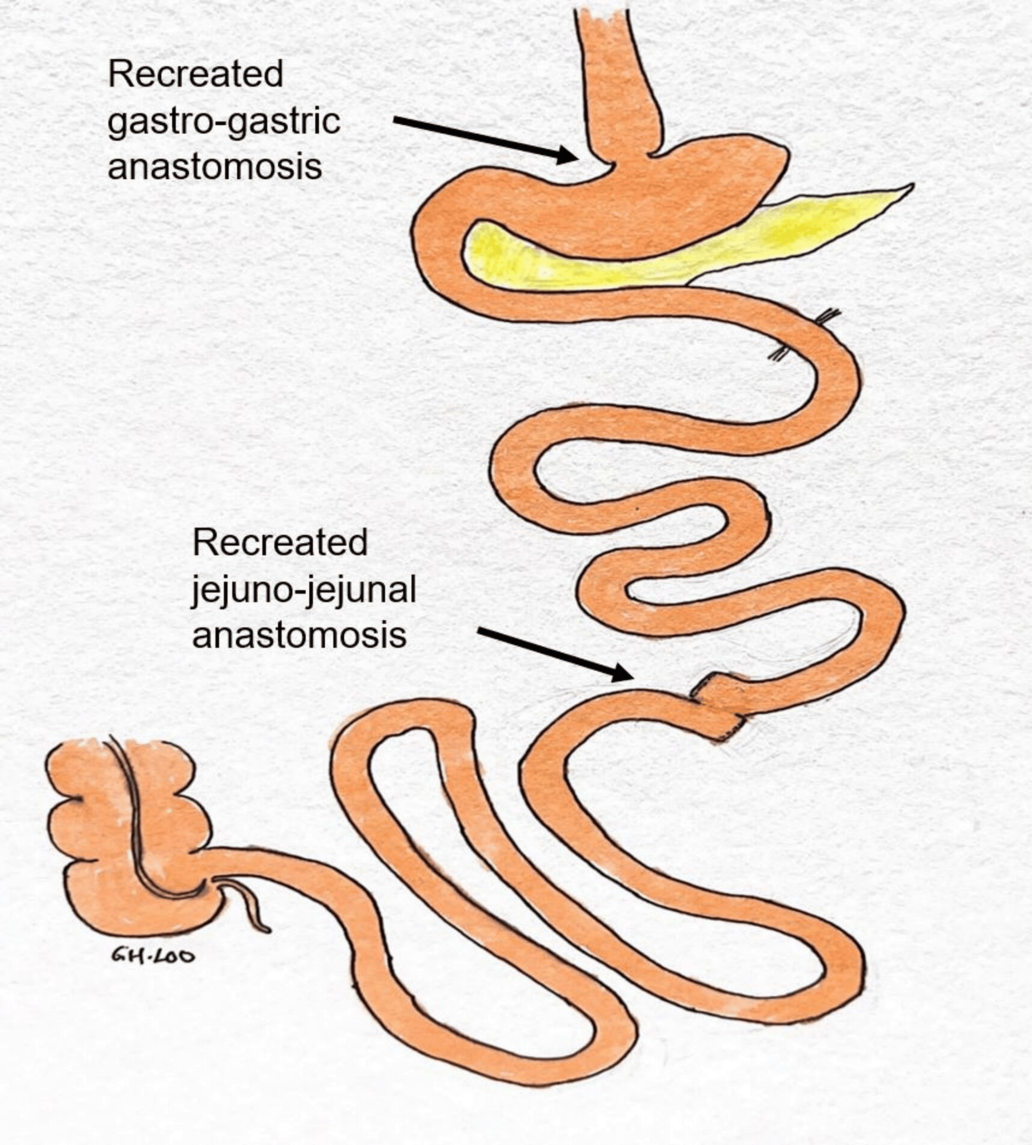
Illustration of the revision of RYGB surgery to normal anatomy RYGB: Roux-en-Y gastric bypass

A multidisciplinary team meeting was held, and it was suggested the best way forward was for a reversal surgery back to normal anatomy, after the optimization of her physical condition. After two weeks of prehabilitation, her weight improved to 40 kg, and she was brought to the operating theater. Intraoperatively, the total small bowel length was 565 cm (from the duodenojejunal flexure to the terminal ileum) with the biliopancreatic limb measuring 105 cm and the Roux limb 45 cm in length. Stapled gastro-gastric anastomosis and jejuno-jejunal anastomosis were performed, reversing the original RYGB (Figure 1).

A methylene blue leak test was performed to ensure the gastro-gastric anastomosis was intact. An edited video of the operative steps is included below (Video 1). 

**Video 1:** Operative procedural steps

She recovered and was discharged on postoperative day 5. During her last follow-up, her latest weight was 45 kg, and her T2DM was still in remission. Her serum albumin and electrolytes remained within the normal range during the follow-up, and she was tolerating a normal diet. 

## Discussion

Laparoscopic reversal of RYGB to normal anatomy is a complex procedure. The decision was often undertaken in patients experiencing complications or failure of the initial bariatric procedure. Among the indications for reversal according to a systemic review by Shoar et al. which included 100 patients were malnutrition (12.8%), severe dumping syndrome (9.4%), postprandial hypoglycemia (8.5%), and excessive weight loss (8.5%). Other factors included marginal ulcer, abdominal pain, intractable nausea and vomiting, protein deficiency recalcitrant hypocalcemia, stricture, etc. [8].

Prior research has established the decline in nutritional status following RYGB. The condition arises due to decreased food consumption or increased excretion caused by the restructuring of the gastrointestinal system. The findings from a retrospective review of patients who underwent RYGB indicate the presence of micronutrient deficiencies, leading to anemia in a range of 20-49% of these individuals. One report from Korea suggested that malnutrition can be attributed not only to the extended use of bypass limbs but also to insufficient food consumption due to postprandial pain [9]. Others suggested the length of the common intestinal and Roux limbs is inversely related to determining the degree of malabsorption, whereas a meta-analysis by Stefanidis et al. suggested a Roux length of 100-150 cm is adequate for patients with a BMI of 50 kg/m^2^ [10]. However, in our case, the patient's preoperative BMI and Roux limb were 27.6 and 45 cm, respectively. According to our local clinical practice guidelines, the bariatric approach may be considered a non-primary alternative for poorly controlled T2DM or metabolic syndrome in individuals with a BMI of >27.5 kg/m^2^; hence, in our patient, it was borderline indicative for bypass surgery. Furthermore, she wasn't given the opportunity for other means of weight loss prior to surgery. The poor patient selection to reverse her metabolic condition may have been attributed to the severity of her malnutrition. Another possibility of her severe malnutrition could have been attributed to her de novo ulcerative colitis post-bariatric surgery; previously, there has been an association between RYGB and the risk of developing Crohn's disease (CD) [11,12].

For a small group of patients, reversal of RYGB remains the only viable option after exhausting medical means of management. In our case, our patient developed severe malnutrition with anemia, which required a reversal of RYGB to normal anatomy [13].

We would like to highlight that preoperative management prior to reversal surgery to normal anatomy following RYGB in a severely malnourished patient is crucial for a successful reversal. It demands a comprehensive approach which includes the assessment of the patient's nutritional status, including serum albumin, vitamin levels, and electrolytes. We recommend initiating parenteral nutrition in patients who cannot tolerate oral intake adequately to optimize surgical readiness. Our patient was started on total parenteral nutrition initially at 10 kcal/kg to prevent refeeding syndrome which was progressively increased to 18 kcal/kg with a soft diet intake. Despite starting parenteral nutrition with low calories, our patient initially developed refeeding syndrome due to her prolonged malnourished state. Due to her low albumin level and pedal edema, we also supplemented her IV human albumin 25% in 250 cc for three days to increase her intravascular oncotic pressure.

Other preoperative strategies involved in managing such a condition include the inclusion of multidisciplinary teams consisting of bariatric surgeons, dietitians, endocrinologists, and other specialists as needed to optimize preoperative management and plan for revision surgery. Surgery should be undertaken when the patient's condition has been stabilized to reduce postoperative complications. By employing these strategies, healthcare providers can optimize preoperative management and revision to normal anatomy after RYGB, improving patient outcomes and quality of life.

The complication rate of RYGB reversal greatly exceeds the reported rates for other types of revisional bariatric surgery, which ranges from 10% to 30% [14]. Vilallonga et al., in particular, illustrated one of the risks of such a complex procedure. The authors reported three anastomotic leaks in their early cases [15]. Ma et al. mentioned that 10% of their patients had gastro-gastric anastomotic leaks which were likely related to the compromised blood supply following dissection and mobilization of the gastric pouch and remnant [16].

In our case, the patient recovered well postoperatively. She was started on nourishing fluid on day 2 after the operation and discharged on day 5. Prior to discharge, she underwent a fluoroscopic study to ensure anastomosis patency. HbA1c upon discharge was 5.4%. In the event our patient regains her initial weight pre-surgery, we could offer her an intragastric balloon which provides 6-15% weight loss compared to only lifestyle interventions which provide 1-5% weight loss.

## Conclusions

The decision to reverse RYGB to normal anatomy should be carefully planned in patients experiencing severe complications such as malnutrition and oral intolerance. Surgical expertise and thorough evaluation of outcomes are crucial in achieving favorable results. This case adds to the existing literature supporting the efficacy of reversal surgery in improving patient outcomes following complications of gastric bypass procedures.
